# Macroevolutionary patterns of ultraviolet floral pigmentation explained by geography and associated bioclimatic factors

**DOI:** 10.1111/nph.13921

**Published:** 2016-03-14

**Authors:** Matthew H. Koski, Tia‐Lynn Ashman

**Affiliations:** ^1^ Department of Biological Sciences University of Pittsburgh Pittsburgh PA 15260 USA; ^2^ Present address: Department of Biology University of Virginia Charlottesville VA 22904 USA

**Keywords:** abiotic selection, biogeography, floral evolution, flower color, phylogenetic comparative methods, *Potentilla*

## Abstract

- Selection driven by biotic interactions can generate variation in floral traits. Abiotic selection, however, also contributes to floral diversity, especially with respect to patterns of pigmentation. Combining comparative studies of floral pigmentation and geography can reveal the bioclimatic factors that may drive macroevolutionary patterns of floral color.
- We create a molecular phylogeny and measure ultraviolet (UV) floral pattern for 177 species in the Potentilleae tribe (Rosaceae). Species are similar in flower shape and visible color but vary in UV floral pattern. We use comparative approaches to determine whether UV pigmentation variation is associated with geography and/or bioclimatic features (UV‐B, precipitation, temperature).
- Floral UV pattern was present in half of the species, while others were uniformly UV‐absorbing. Phylogenetic signal was detected for presence/absence of pattern, but among patterned species, quantitative variation in UV‐absorbing area was evolutionarily labile. Uniformly UV‐absorbing species tended to experience higher UV‐B irradiance. Patterned species occurring at higher altitudes had larger UV‐absorbing petal areas, corresponding with low temperature and high UV exposure.
- This analysis expands our understanding of the covariation of UV‐B irradiance and UV floral pigmentation from within species to that among species, and supports the view that abiotic selection is associated with floral diversification among species.

## Introduction

Flower color, and color patterns in particular, can mediate plant–pollinator interactions (Medel *et al*., 2003; Grossenbacher & Stanton, 2014; Koski & Ashman, 2014; Muchhala *et al*., 2014), prompting many to focus on pollination as the most likely ecological process to generate flower color diversity (Rausher, 2008; Smith *et al*., 2008; van der Niet & Johnson, 2012; Grossenbacher & Stanton, 2014; Muchhala *et al*., 2014). Indeed, shifts in pollination have been associated with divergence in floral phenotypes among taxa, supporting pollinators as drivers of floral evolution (reviewed by van der Niet & Johnson, 2012). Nonetheless, there is strong evidence from microevolutionary studies that abiotic factors contribute flower color diversity as well (Schemske & Bierzychudek, 2001; Warren & Mackenzie, 2001; Coberly & Rausher, 2003). Such selection can even lead to broad‐scale geographic patterns of flower pigmentation (Arista *et al*., 2013; Koski & Ashman, 2015a). However, whether abiotic factors also contribute to diversity in floral traits across species has not been determined.

**Figure 1:**
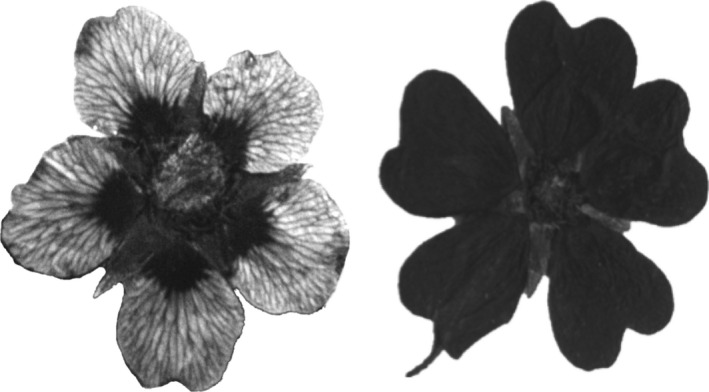
UV images of a species with a UV bullseye floral phenotype (left, *Potentilla eriocarpa*) and a uniformly UV‐absorbing floral phenotype (right, *Potentilla evestita*). Flowers are from pressed herbarium specimens and both are uniformly yellow in the human‐visible spectrum.

An abiotic perspective may be particularly relevant to the evolution of floral pigmentation in the ultraviolet (UV) spectrum (Fig. 1) for several reasons. First, selection may act indirectly on flower color via the pleiotropic action of genes that confer vegetative pigmentation (reviewed by Rausher, 2008; Wessinger & Rausher, 2012). Specifically, products of the flavonoid biosynthetic pathway that protect vegetative tissues from UV‐B irradiance, temperature, and/or drought stress also underlie UV‐absorbing pigments in floral tissue (Harborne & Nash, 1984; Rausher, 2008; Wessinger & Rausher, 2012). Second, abiotic factors can mediate selection directly on UV floral pigmentation because light reflected from petals can affect the floral environment for pollen and ovules (Koski & Ashman, 2015a). For example, in *Argentina anserina*, larger areas of UV‐absorbing pigmentation on petals protect pollen from UV damage, and this is proposed to underlie the correlation between UV area and UV incidence across latitudes (Koski & Ashman, 2015a). Likewise, at a regional scale, increased pigmentation is correlated with increasing UV irradiance with altitude in this species (Koski & Ashman, 2015b). Although studies in a single species are compelling demonstrations of the strength of UV‐B irradiance as a selective force on flower color patterns, the question remains as to whether these latitudinal and altitudinal associations also occur across species.

Moreover, bioclimatic variables other than UV‐B exposure correlate with altitude and latitude. For example, temperature and precipitation decrease with increasing latitude, and temperature decreases while clear‐sky UV exposure increases with increasing altitude (reviewed by Körner, 2007). In temperate regions, precipitation also increases with altitude (Körner, 2007). Thus, while geography is a good predictor of abiotic conditions, associating phenotypic variation directly with bioclimatic variables can pinpoint the drivers of geographic variation (e.g. Galen, 2000; Arista *et al*., 2013; Bontrager & Angert, 2016; Koski & Ashman, 2015a). For example, Arista *et al*. (2013) found that a latitudinal trend in a flower color polymorphism was explained largely by sunlight hours, while much less of the variation was explained by precipitation. Thus, it is important for our general understanding of the selective forces that act on floral phenotype to consider both geographic and bioclimatic variables.

When assessing correlations between traits and ecological drivers across species, one also needs to account for phylogenic relationships because related species can resemble one another and occupy similar niches (Felsenstein, 1985; Losos, 2008; Revell, 2010) as a result of shared ancestry. However, most studies find that phylogenetic relatedness does not structure patterns of variation in human‐visible flower color across phylogenies (Smith *et al*., 2008; Muchhala *et al*., 2014; Gómez *et al*., 2015), suggesting that divergence in flower color is not strongly constrained by shared evolutionary history. Floral UV patterns, however, while highly variable among closely related species (e.g. Rieseberg & Schilling, 1985; Naruhashi & Ikeda, 1999), have not been evaluated from this perspective in any group. Thus, our understanding of the ecological correlates with UV floral pattern at a macroevolutionary level will be improved by determining the degree to which evolutionary history structures phenotypic variation across species, and whether bioclimatic factors are responsible for geographic patterns of floral pigmentation, with emphasis on the abiotic forces demonstrated to act at the microevolutionary level.

The Potentilleae tribe (Rosaceae) consists of herbs and shrubs that vary widely in their latitudinal and altitudinal ranges, that is, from the lowland coastal zone to the high‐altitude tundra. Most species possess yellow five‐merous flowers in the human‐visible spectrum but may be either patterned (UV bullseye) or uniformly UV‐absorbing (Fig. 1; Harborne & Nash, 1984; Naruhashi & Ikeda, 1999). Additionally, among the patterned species there is quantitative variation in the size of UV‐absorbing portion of the petal (Naruhashi & Ikeda, 1999). We assessed geographic and bioclimatic patterns of UV floral pigmentation in species of the Potentilleae tribe. Based on our previous work (Koski & Ashman, 2015a,b), we predicted that species growing at lower latitude and/or higher altitude have larger proportions of their petals absorbing UV, and a larger area of UV absorption is associated with higher UV irradiance. In addition, based on published work evaluating the fitness of pigmented and nonpigmented floral morphs, we predict that floral UV pigmentation is more extensive in species that experience higher temperature (Coberly & Rausher, 2003) and/or lower precipitation (Schemske & Bierzychudek, 2001; Warren & Mackenzie, 2001). To test these predictions, we evaluated the extent of interspecific variation in UV pattern of flowers and determined the degree to which evolutionary history contributes to the presence of UV pattern and quantitative variation in the extent of UV pigmentation. We then determined whether latitudinal or altitudinal distribution predicts UV pattern variation, and subsequently what abiotic factors (temperature, precipitation, UV‐B irradiance) underlie geographic patterns across taxa.

## Materials and Methods

### System

The Potentilleae tribe (Rosaceae, Rosoideae) consists of four subtribes and 19–20 genera (Soják, 2008). One of the largest genera in the group, *Potentilla*, has 300–430 extant species that occupy temperate and arctic distributions. All species' flowers are radially symmetrical, and most often yellow (but can be white, or, rarely, red) in the human‐visible color spectrum. Species with UV‐patterned and uniformly UV‐absorbing flowers are documented in *Potentilla* (Harborne & Nash, 1984; Naruhashi & Ikeda, 1999) but the presence of pattern was previously unknown in other genera included in this study (*Horkelia*,* Ivesia*,* Drymocallis*,* Sibbaldia*,* Sibbaldiopsis*). In species for which the biochemistry of floral pigmentation has been explored (various *Potentilla*,* Dasiphora* and *Argentina*), UV absorption is manifested by flavonoid compounds (e.g. quercetin) while yellow pigmentation is the result of carotenoids, and red pigmentation is underlain by cyanidins (Harborne & Nash, 1984). Of the taxa for which pollination has been assessed (*Potentilla erecta* (Lázaro *et al*., 2009); *Potentilla pulcherrima* (Burkle & Irwin, 2010); *Potentilla recta* (McIver & Erickson, 2012); *Potentilla gracilis* (McIver & Erickson, 2012); *Argentina anserina* (Koski & Ashman, 2014, 2015b)), flowers are pollinated by a variety of small bees and flies of several genera.

### Phylogeny

#### Plant material

We obtained leaf material of 130 species from the Royal Botanic Garden Edinburgh Herbarium (E), Carnegie Mellon Museum of Natural History Herbarium (CM), Rancho Santa Ana Botanic Garden Herbarium (RSA), Harvard University Herbaria (A), University and Jepson Herbaria (UC & JEPS), and Rocky Mountain Herbarium (RM). We provide accession information (herbarium voucher, collector, locality, GenBank reference) in Supporting Information Table S1. We followed accepted species names from The Plant List (theplantlist.org).

#### DNA extraction, PCR and sequencing

We extracted genomic DNA from 1–2 mg of dried herbarium leaf tissue using the CTAB method (Doyle, 1987). We sequenced two nuclear (internal transcribed spacer (ITS); external transcribed spacer (ETS)) and one chloroplast (*trn*L‐F) region for 126, 122 and 86 specimens, respectively. We obtained the full ITS region using the ITS‐1 (Urbatsch *et al*., 2000) and ITS4 (White *et al*., 1990) primers. For some taxa (25%), we used internal primers ITS2 (White *et al*., 1990) and ITS3b (Baum *et al*., 1994) to obtain the full ITS region. We sequenced ETS using ETS1 and IGS6 primers (Oh & Potter, 2005) and the partial *trn*L‐F region using trnL^UAA^3′ and trnF^GAA^ primers (Taberlet *et al*., 1991). Annealing temperatures were 50, 54 and 51°C for ITS, ETS and *trn*L‐F reactions, respectively.

We confirmed PCR products on a 1% agrose gel, purified them using Exo‐SAP (Affymetrix/USB, Cleveland, OH, USA), and sequenced them on an ABI 3730XL DNA analyzer (Applied Biosystems/Life technologies, Carlsbad, CA, USA). We constructed consensus sequences of forward and reverse reads using sequencher 5.3 (Gene Codes Corp., Ann Arbor, MI, USA).

To augment our data with additional species (*n *=* *50) and sequences (*n *=* *170), we obtained published sequence from GenBank (Erikkson *et al*., 2003; Dobeš & Paule, 2010; Töpel *et al*., 2011). All GenBank accession numbers are in Table S1.

#### Phylogenetic reconstruction

We aligned ITS, ETS and *trn*L‐F sequences separately using clustalw (Thompson *et al*., 1994) and manually edited gaps in mega v.5.2.2 (Tamura *et al*., 2007). We deleted three sites of ambiguous alignment in the *trn*L‐F matrix (272‐313, 388‐406, 518‐541). We verified the best nucleotide substitution model for each gene using jmodeltest2 (Darriba *et al*., 2012), all of which conformed to a GTR + G + I model.

We concatenated the three regions for generation of backbone trees for use in comparative analyses. The final sequence matrix included 183 taxa. We constructed the phylogeny with beast v.1.8.1 (Drummond *et al*., 2012). Each gene was modeled with a log‐normal relaxed clock, and we used a birth–death process of speciation. We ran two separate 400 000 000 generation Markov chain Monte Carlo (MCMC) chains, logging parameters every 1000. We time‐calibrated the phylogeny by setting the node ages of two previously described clades – the *Argentina* clade (21.2–32 million yr ago (Ma)) and the *Drymocallis* clade (2.3–6.7 Ma) – with uniform normal distributions. Ages were based on estimates of divergence time from a fossil‐calibrated phylogeny by Dobeš & Paule (2010). The *Argentina* clade included (all genus *Potentilla*) *A. anserina*,* P. anserinoides*,* P. lignosa*,* P. fulgens*,* P. lineata*,* P. microphylla*,* P. stenophylla*,* P. festiva*,* P. peduncularis*,* P. cardotiana*,* P. leuconota*, and the *Drymocallis* clade included *D. fissa*,* D. convallaria*,* D. deseretica*,* D. glabrata*,* D. glandulosa*,* D. lactea* var. *austiniae*,* D. rupestris*, and *Potentilla drummondii* grouped in the *Drymocallis* clade in preliminary analyses).

We confirmed convergence of the two MCMC chains, and assessed effective sample sizes (ESSs) for parameter estimates in Tracer. ESSs for ITS, ETS, *trn*L‐F, and coalescent tree likelihoods were 1264, 435, 283 and 1065, respectively. For computational resource purposes, before analyses in beast, we thinned the combined log file by sampling every 3800 chains in logcombiner and generated an ultrametric maximum clade credibility (MCC) tree, and a set of posterior trees with 10 000 burn‐in trees using treeannotator v.1.8.1 (Drummond *et al*., 2012), resulting in 200 527 posterior trees. We subsampled 200 random trees from the posterior distribution for downstream comparative analyses to account for phylogenetic uncertainty. Results from analyses across this set of trees were congruent with those obtained from analyses using the consensus phylogeny.

### Phenotypic, geographic, and bioclimatic data

#### Floral phenotypes

We measured UV proportion (UVP; the proportion of the petal that absorbs UV) from 563 specimens of 177 species (range: 1–10 per taxon; mean = 3.2). We photographed flowers in the UV spectrum, and measured UVP following Koski & Ashman (2013). UV pattern has been reliably scored from herbarium samples in other studies (Horovitz & Cohen, 1972), and pressing flowers does not affect UVP (Koski & Ashman, 2013). We measured one to two petals on flowers with adaxially facing petals. If multiple flowers on a plant were available, we measured all and averaged measurements. When two separate plants with measurable flowers were preserved on a sheet, they were treated as separate specimens. For one species, *P. peduncularis*, we analyzed UVP from an image published by Naruhashi & Ikeda (1999). For *P. anserinoides*, we used five field‐collected samples (Koski & Ashman, 2015a), and for *P. anserina*, we analyzed two dried flowers from glasshouse‐grown plants. We were unable collect phenotypic data for five species included in the molecular phylogeny (*Ivesia arizonica* var. *saxosa, Horkelia fusca* var. *pseudocapitata, Potentilla humifusa, Potentilla neumaniana, Potentilla parvifolia, Sibbaldia parviflora*). These species were subsequently trimmed from the phylogeny for comparative analyses. We averaged UVP for each species (Table S2) for phylogenetic analyses.

In addition to the quantitative variable of UVP, we scored UV pattern as a discrete binomial character (e.g. Harborne & Nash, 1984) where ‘UV patterned’ flowers were those with a detectable amount of the petal apex reflecting UV (UVP < 0.95) and uniformly UV‐absorbing flowers were those with UVP ≥ 0.95.

To account for potential effects of environmental variation on UVP, we sampled more specimens from taxa with larger range areas, latitudinal ranges, and altitudinal ranges (range area vs samples per species, *r *=* *0.23, *P *=* *0.003, *n *=* *161; latitudinal range vs samples per species, *r *=* *0.26, *P < *0.001 *n *=* *167; altitudinal range vs samples per species, *r *=* *0.34, *P *<* *0.0001, *n *=* *166; data not shown). To assess whether the degree of variation in UVP was greater among, rather than within, taxa for the species with multiple samples (*n *=* *136), we partitioned variance among samples using ANOVA with a main effect of species identity.

While the focus of this study was UV floral pigmentation, we also recorded human‐visible flower colors to assess whether certain human‐visible flower colors were associated with UV pigmentation pattern. We scored human‐visible flower from descriptions on herbarium labels and online images. Categories for human‐visible flower color were yellow, white, and red.

#### Bioclimatic data

We gathered locality data for each species from the Global Biodiversity Information Facility (GBIF.org) and additional databases (SEINnet, http://swbiodiversity.org; Consortium of the Pacific Northwest Herbaria, pnwherbaria.org; Tropicos, http://tropicos.org; Royal Botanic Gardens Edinburgh, http://rbge.org.uk/bgbase/vherb; and University of Vienna Herbarium, http://herbarium.univie.ac.at/database). We eliminated duplicate data points and localities outside of the natural or naturalized range (e.g. botanic gardens). In total we compiled 374 829 localities with a mean of 2118 per species. We used DIVA‐GIS and the WorldClim dataset (Hijmans *et al*., 2005) to obtain latitude and altitude and bioclimatic data (annual mean temperature, annual mean precipitation) for each georeferenced specimen. Additionally, we extracted mean annual UV‐B irradiance in DIVA‐GIS using data from Beckmann *et al*. (2014). We averaged temperature, precipitation, and UV‐B irradiance for each species (Table S2).

### Effect of phylogeny on distribution of UV pattern across species

**Figure 2:**
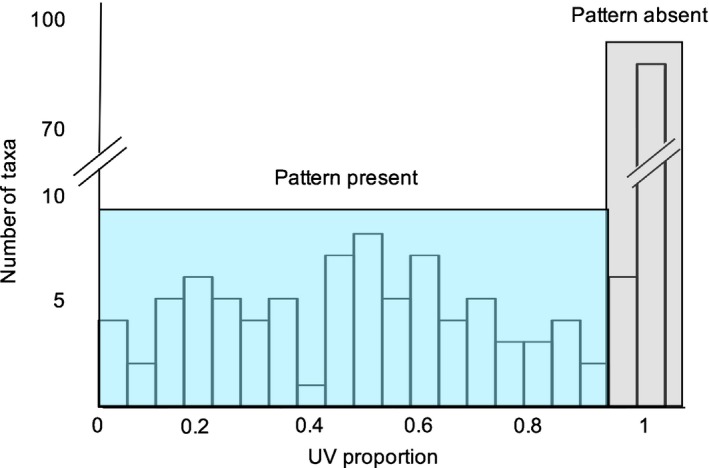
Variation in UV proportion (UVP) for 177 species in Potentilleae measured on UV images of herbarium samples. Nearly half (*n *=* *86) of the taxa displayed UV‐reflective petal tips (UVP < 0.95) while the remainder (*n *=* *91) displayed no UV reflection, and thus no UV pattern (UVP > 0.95).

We tested for phylogenetic signal (the degree to which closely related species resemble one another more than distantly related species) for the presence/absence of UV pattern and geographic (latitude, altitude) and bioclimatic (temperature, precipitation, UV‐B irradiance) parameters for all 177 species for which we scored UVP. For ‘UV patterned’ taxa only (*n *=* *86; Fig. 2), we estimated phylogenetic signal for quantitative variation in UVP and geographic and bioclimatic parameters. A similar two‐stage approach (with both discrete and continuous response variables) has been implemented in other systems with a bimodal phenotypic distribution (e.g. degree of cooperation in wasps; Sheehan *et al*., 2015). We calculated Blomberg's *K* (Blomberg *et al*., 2003) for quantitative variables (UVP, latitude, altitude, temperature, precipitation, UV‐B irradiance), where *K* < 1 suggests that more related species resemble each other less than expected under a Brownian motion (BM) model of evolution, and *K* > 1 indicates they are more similar. We calculated *K* with the ‘multiPhylosignal’ function (picante; Kembel *et al*., 2010) and tested whether it was significantly greater than zero (i.e. phylogenetic relatedness explains none of the variation) with 999 randomizations. For the presence/absence of UV pattern, we calculated Pagel's *λ* and tested whether it was > 0 using the ‘phylosig’ function in the R package phytools. Pagel's *λ* is a branch scaling parameter where *λ* = 0 indicates that the tree topology does not structure trait variation, while *λ* = 1 indicates that the trait is distributed on the tree in accordance with BM. The test of whether *λ* is significantly > 0 is performed by comparing the log‐likelihood of the fitted *λ* with that of *λ* = 0 using a log‐likelihood ratio test. We report mean ± SE of *K* and *λ*, and average *P*‐values calculated across the 200 random posterior trees to account for phylogenetic uncertainty.

### Geographic and bioclimatic association with UV floral pattern

We evaluated whether the presence/absence of UV pattern was associated with latitude and/or altitude (hereafter, geographic model), and temperature, precipitation and/or UV‐B irradiance (hereafter, bioclimatic model) with generalized estimating equations (GEEs) with a binomial response variable (UV pattern present = 0, pattern absent = 1) using the compar.gee function in the ape package (Paradis & Claude, 2002). We used a binomial distribution with a logit link function and the correlation structure was the phylogeny with branch lengths transformed to 1. To facilitate comparison between the effects of predictor variables on the presence/absence of UV pattern, we standardized predictor variables by first calculating the phylogenetic mean, subtracting that from the value for each species, and dividing by the standard deviation of the phylogenetic mean using the phyl.vcv function in phytools (R code by L. Revell online; https://stat.ethz.ch/pipermail/r-sig-phylo/2015-February/003885.html). GEEs were performed on a set of 200 trees, and we report the average ± SE of parameter estimates and significance values across trees.

To address whether geographic and bioclimatic variables predicted quantitative variation in UVP among the UV‐patterned taxa alone, we conducted phylogenetic least‐squares regression (PGLS). We used the phylolm function in the phylolm package in R with both BM and Ornstein–Uhlenbeck (OU) phylogenetic correlation structures (Tung Ho & Ane, 2014). Under the BM correlation structure, traits are assumed to evolve under a model of random genetic drift, while OU incorporates a stabilizing selection parameter (*α*) that constrains phenotypic variation to an optimum, that is, it assumes selection influences evolution of the trait of interest (Butler & King, 2004). We compared Akaike information criterion values between BM and OU models and conducted log‐likelihood ratio tests to determine the best fit. We trimmed species from a set of 200 trees such that only those with UV pattern (UVP < 0.95) were present. Additionally, to visualize the relationships between UVP and each predictor variable alone, while accounting for phylogenetic correlation, we generated slopes from linear regressions of the phylogenetic independent contrast (PIC) for UVP on the PIC for each predictor variable separately with intercepts set to zero (Felsenstein, 1985; Garland *et al*., 1992) across the 200 trees with the ‘pic’ function in the ape package.

## Results

### Phylogeny

The phylogeny based on a 1920 bp (ITS = 792, ETS = 544, *trn*L‐F = 584) sequence matrix of 183 species in the Potentilleae tribe was topologically congruent with published phylogenies but included 67 species not previously included (Dobeš & Paule, 2010; Töpel *et al*., 2011). The MCC topology with posterior probabilities is provided in Fig. S1. Of 182 internal nodes, 65% had posterior probability (PP) support of > 0.50, 50% had PP* *>* *0.75, and 40% had PP > 0.90 (Fig. S1). The presence of nodes with little support warranted the use of multiple trees from the posterior distribution for comparative analyses.

The largest and least diverged group which included *Potentilla* was consistent with other studies (PP* *=* *0.92). *Potentilla* included the *Horkelia* /*Ivesia* clade (PP* *=* *0.94) (Fig. S1). *Potentilla biennis*,* Potentilla norvegica*,* Potentilla rivalis*,* Potentilla newberryi*, and *Potentilla intermedia* were within the *Horkelia* /*Ivesia* clade (*P *=* *0.94). A number of *Potentilla* that are morphologically similar to *Argentina* grouped with *Argentina* (*P *=* *1), as expected based on previous phylogenies (Dobeš & Paule, 2010). Additionally, *P. drummondii* grouped with the monophyletic *Drymocallis* clade (*P *=* *0.97; Fig. S1).

Within *Potentilla* there was support (*P *=* *0.96) for the previously described Alba clade (Fig. S1) (Töpel *et al*., 2011). The previously described Fragarioides clade was also recovered (*P *=* *0.96; Fig. S1), but included two species that were within the Töpel *et al*. (2011) Alba clade (*P. articulata*,* P. biflora*). The Reptans clade (Dobeš & Paule, 2010; Töpel *et al*., 2011) and was also recovered (PP* *=* *0.92; Fig. S1).

### UV floral pattern

Variation in the area of the petal that absorbed UV reflected the full possible range from 0 (fully UV‐reflective petals) and 100% (fully UV‐absorptive petals) (Fig. 2). Eighty‐six species had UV pattern (UVP < 0.95), while 90 were uniformly UV‐absorbing (UVP > 0.95). Species with UV pattern on petals were observed in *Potentilla*,* Ivesia*,* Horkelia* and *Duchesnea*, but not *Drymocallis*,* Dasiphora* or *Sibbaldia*. Within *Potentilla*, the Alba clade was the only one in which all species lacked UV pattern. The majority (80%) of UVP variation was among, rather than within, taxa (ANOVA, *F*_134,349_ = 10.14, *P *<* *0.0001).

Most species included in the phylogeny have yellow flowers in the human‐visible spectrum (79%, *n *=* *140). Thirty species (17%) have white flowers, while seven (4%) have red flowers (restricted to *Potentilla*). UV pattern was observed on flowers with all of these human‐visible colors, but of the 86 taxa with UV pattern, 94% had yellow flowers. Of the yellow‐flowered species, 58% were UV‐patterned, while 7% and 43% of the white‐flowered and red‐flowered species were patterned, respectively.

### Geographic and bioclimatic parameters

The range of georeferenced localities obtained per taxon was wide (1–80 979), reflecting the fact that some species have very restricted ranges (e.g. *Potentilla deorum* endemic to Mt Olympus, Greece) whereas others have a global distribution (e.g. *A. anserina*). The average latitude of species’ range spanned from the tropical zone (21.07°N; Mexican endemic, *Potentilla ranunculoides*) to the arctic zone (76.49°N; circumpolar, *Potentilla pulchella*), but most taxa are of temperate distribution (among all taxa: mean latitude = 43.04°N). Average altitude ranged from 32 to 4395 m with a mean of 1705 m. Annual temperature was highly variable (range: −14.48–18.14°C, mean = 4.89°C), as was annual rainfall (range: 117–2780 mm, mean = 729.2 mm) and annual UV‐B irradiance (range: 1072.6–6013.4 J m^−2^ d^−1^, mean = 3003 m^−2^ d^−1^). Mean species geographic and bioclimatic parameters are provided in Table S2.

### Effect of phylogeny on distribution of UV pattern across species

**Figure 3:**
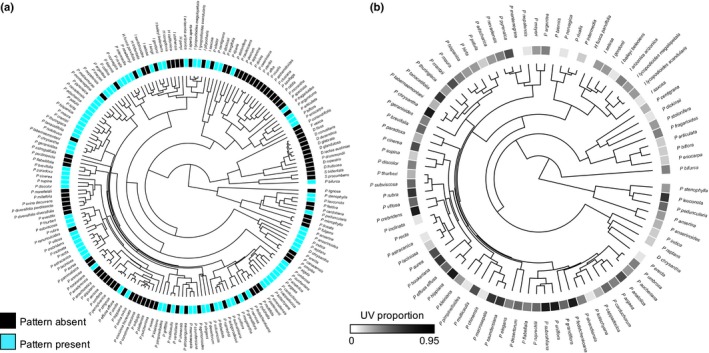
(a) Phylogenetic distribution of the presence (UVP < 0.95) and absence (UVP > 0.95) of floral UV pattern in 176 species in Potentilleae. (b) Phylogenetic distribution of UV proportion for species with UV pattern (UVP < 0.95) for 86 species in Potentilleae. Trees are maximum clade credibility trees created in beast from combined nuclear (internal transcribed spacer (ITS); external transcribed spacer (ETS)) and chloroplast (*trn*L‐F) sequences.

Pagel's *λ* was significantly > 0 for the presence/absence of UV pattern displayed (Table 1; Fig. 3a). Similarly, *K* was > 0 for species mean altitude and bioclimatic variables (temperature, precipitation and UV‐B irradiance) when considering all taxa (Table 1). For species with UV pattern only, *K* was > 0 for UVP, but this was only marginally significant (Table 2; Fig. 3b), while *K* was significantly > 0 for all geographic and bioclimatic parameters (Table 2). Given that common ancestry affected the distribution of geographic, bioclimatic and phenotypic measurements across the phylogeny, we proceeded with phylogenetically informed tests of ecological associates with UV pattern variation.

**Table 1:** Mean and SE of Blomberg's *K* or Pagel's *λ* for the presence or absence of UV pattern, geographic features (latitude and altitude), and bioclimatic conditions (temperature, precipitation, UV irradiance) for 176 species in Potentilleae measured on 200 phylogenetic trees from the posterior tree distribution

Trait or feature	*K* or *λ*	SE	*P*
UV pattern (present/absent)	**0.602**	0.011	**0.042**
Latitude	0.115	0.004	0.078
Altitude	**0.116**	0.004	**0.046**
Temperature	**0.148**	0.005	**0.047**
Precipitation	**0.176**	0.005	**0.018**
UV‐B irradiance	**0.140**	0.004	**0.039**

Average *P‐*values across all trees are shown and factors with *K* or *λ* > 0 are in bold. Pagel's *λ* was used to estimate phylogenetic signal for UV pattern. For all other traits, Blomberg's *K* was used.

**Table 2:** Mean and SE of Blomberg's *K* for UV proportion, geographic features, and bioclimatic conditions for 86 species in Potentilleae measured on 200 phylogenetic trees from the posterior tree distribution

Trait or feature	K	SE	*P*
UV proportion	0.168	0.005	0.069
Latitude	**0.201**	0.006	**0.046**
Altitude	**0.205**	0.006	**0.054**
Temperature	**0.179**	0.005	**0.037**
Precipitation	**0.349**	0.009	**0.013**
UV‐B irradiance	**0.245**	0.007	**0.032**

Average *P‐*values across all trees are shown and factors with *K* > 0 are in bold.

### Is presence/absence of UV pattern predicted by geography or bioclimate?

Based on GEE models, latitude and altitude did not predict the presence/absence of UV pattern, although both parameters were positive and the effect of altitude was stronger than latitude (Table 3). The bioclimatic model indicated a marginally significant positive effect of UV irradiance (*P *=* *0.068) on the presence/absence of UV pattern; that is, species with uniform UV absorption tended to exist in environments that experience higher UV‐B irradiance than UV‐patterned species. The effects of temperature and precipitation were negative and positive, respectively, but neither was significant.

**Table 3:** The effect of geographic range (latitude and altitude) and bioclimatic parameters (temperature, precipitation, UV irradiance) on the presence/absence of UV pattern on flowers of 177 Potentilleae species as determined by generalized estimating equations accounting for phylogenetic correlation

Model	Parameter	Estimate	SE	*P*	QIC
Geographic	Intercept	0.524	0.024	0.104	262.322
	Latitude	0.961	0.034	0.563	
	Altitude	2.915	0.032	0.107	
Bioclimatic	Intercept	0.259	0.026	0.190	257.432
	Temperature	−2.764	0.039	0.103	
	Precipitation	1.653	0.032	0.321	
	UV‐B irradiance	2.831	0.036	0.068	

Mean and SE of parameter estimates, and mean *P*‐values are from regressions performed on 200 Bayesian posterior trees. Predictor variables were standardized so that the magnitudes of effects are comparable. QIC, Quasilikelihood Information criterion. Phylogenetic degrees of freedom (dfP) = 28.139 for all models.

### Is quantitative variation in UV pattern predicted by geography or bioclimate?

**Figure 4:**
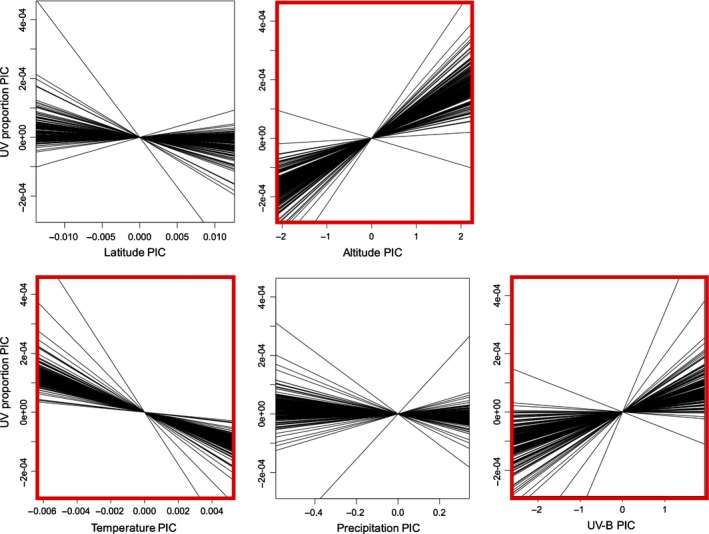
Slopes of UV proportion phylogenetic independent contrasts (UVP PICs) regressed on PICs for geographic and bioclimatic parameters conducted on 200 trees, including only UV‐patterned species (*n *=* *86). Intercepts were set to zero. Geographic and bioclimatic variables that significantly influenced UV proportion in standardized multiple phylogenetic regressions (Table 4) are highlighted in red.

Whether considering either BM or OU phylogenetic correlation structures, altitude emerged as a strong positive predictor of greater UV pigmentation on flowers, and the OU model provided a better fit than a BM model (log‐likelihood ratio test, *P *<* *0.0001; Table 4). Under an OU model, the positive effect of altitude (Table 4; Fig. 4) was 1.4 times stronger than that of latitude, which was only marginally significant (*P *=* *0.06; Table 4). Thus, our prediction that species growing at higher altitudes would have higher UV pigmentation was supported. The prediction that those growing at lower latitudes would have higher UV pigmentation, however, was not supported.

**Table 4:** The effect of geographic range parameters (latitude and altitude) and bioclimatic parameters (temperature, precipitation and UV irradiance) on UV proportion for taxa in Potnetilleae with UV pattern (*n *=* *86) determined by multiple phylogenetic least‐square regressions with both Brownian motion (BM) and Ornstein–Uhlenbeck (OU) correlation structures

Model	Parameter	Estimate	SE	*P*	AIC	Log‐likelihood
Geographic BM	Intercept	0.45	9.40E–04	0.25	37.5	−14.7
	Latitude	1.11E–04	3.50E–05	0.12		
	Altitude	**2.90E–04**	**7.46E–05**	**0.007**		
Geographic OU	Intercept	**0.45**	**1.20E–03**	**< 0.0001**	10.4	−0.2
	Latitude	1.18E–04	3.49E–05	0.06		
	Altitude	**2.85E–04**	**7.36E–05**	**0.009**		
Bioclimatic BM	Intercept	0.45	9.35E–04	0.23	26.7	−8.37
	Temperature	−**2.75E–04**	**5.92E–05**	**0.0002**		
	Precipitation	1.10E–04	2.59E–05	0.07		
	UV‐B irradiance	**1.50E–04**	**3.17E–05**	**0.02**		
Bioclimatic OU	Intercept	**0.47**	**1.66E–03**	**< 0.0001**	1.65	5.17
	Temperature	−**2.70E–04**	**5.93E–05**	**0.0001**		
	Precipitation	1.02E–04	2.60E–05	0.07		
	UV‐B irradiance	**1.40E–04**	**2.18E–05**	**0.04**		

Mean and SE of parameter estimates, and mean *P*‐values are from regressions performed on 200 trees. Significant intercepts and predictor variables from models are in bold. Predictor variables were standardized so that estimates are comparable. Mean alpha parameters for OU models were 4.5E–07 and 4.0E–07 for the geographic and bioclimatic models, respectively. AIC, Akaike information criterion.

Bioclimatic variables predicted UVP variation among taxa with both the BM and OU model, with the OU model providing a better fit (log‐likelihood ratio test, *P *<* *0.0001; Table 4). Temperature had the strongest effect on UVP, while UV‐B irradiance also had an effect (Fig. 4). Specifically, species experiencing lower temperatures and greater UV‐B irradiance had larger areas of UV‐absorptive pigmentation on flowers (Table 4; Fig. 4). For the UV‐patterned species only, altitude was negatively correlated with temperature (*r *=* *−0.28, *P *<* *0.01) and precipitation (*r *=* *−0.21, *P *=* *0.05), and positively correlated with UV (*r *=* *0.84, *P *<* *0.0001). Thus, the effect of altitude from the geographic model is in line with effects of temperature and UV revealed by the bioclimatic model. The prediction that UV‐B irradiance would have a positive effect on UV pigmentation was supported. The negative effect of temperature that we detected was in the opposite direction of our prediction, and the prediction that pigmentation would decrease with precipitation was not supported.

## Discussion

By identifying geographic and bioclimatic factors associated with diversity in floral UV pigmentation in Potentilleae, this is the first study to support the hypothesis that abiotic selection may drive floral diversification among species. Species with uniformly UV‐absorbing flowers tended to grow in areas of higher UV‐B irradiance than those with smaller areas of UV, that is, with UV pattern. Moreover, considering the species for which UV pattern was present, altitude, temperature, and UV‐B irradiance all significantly predicted the extent of UV pigmentation. Namely, species growing at higher altitudes, where temperature is lower and UV‐B irradiance is higher, have greater portions of their petals with UV‐absorbing pigmentation. While pollinator communities may covary with geography and bioclimatic variables and contribute to observed variation, our findings are in accordance with mechanistic studies showing that UV irradiance directly imposes positive selection on UV pigmentation (Koski & Ashman, 2015a). This comparative approach suggests that the abiotic selective processes acting on UV floral pattern at the microevolutionary level may also be responsible for structuring variation at the macroevolutionary level.

The bimodal distribution of UV pigmentation pattern in the Potentilleae tribe, with one mode at an intermediate‐sized UV bullseye and one at uniform UV absorption, is suggestive of two adaptive peaks (e.g. Muir, 2015). For example, a bullseye phenotype may enhance fitness through its mediation of plant–pollinator interactions (e.g. Koski & Ashman, 2014), while uniform UV absorption may enhance fitness via protection from abiotic stressors (e.g. Koski & Ashman, 2015a). Whether selective constraint gives rise to the observed multimodality of UV floral pigmentation should be examined using recently developed models of trait evolution that incorporate OU processes for proportional traits (Muir, 2015). The UV bullseye was detected on species of all human‐visible flower colors present in Potentilleae (yellow, red and white). Observations of multiple diverse floras suggest that yellow flowers are more likely to reflect UV and possess UV pigmentation pattern than white or green flowers (Guldberg & Atsatt, 1975; Inouye & Pyke, 1988; Dyer, 1996). About 58% of the yellow‐flowered species we scored had UV pattern, while only 7% of the white‐flowered species did, lending support to previously observed correlations. This may be driven by biochemical constraint or correlated selection. Whereas we focused on the effects of extrinsic abiotic factors on UV pigmentation, an interesting next step will be to determine the degree to which trait correlations structure UV pattern variation.

A number of studies have scored UV pattern for closely related species (Horovitz & Cohen, 1972; Harborne & Nash, 1984; Rieseberg & Schilling, 1985; Naruhashi & Ikeda, 1999), but this study was able to assess this variation in a phylogenetic framework. More closely related species tend to be more similar for the presence/absence of UV pattern than those that are more distantly related. This is especially apparent in the *Drymocallis* and Alba clades, in which all taxa display only uniformly UV‐absorbing flowers (Fig. 3a). Quantitative estimates of UV pigmentation for taxa with UV pattern, on the other hand, only displayed marginally significant Blomberg's *K*, suggesting that the UV pattern scored in this manner is not highly structured by shared evolutionary history (Fig. 3b). As Blomberg's *K* statistic does not covary with tree size (Blomberg *et al*., 2003), we do not expect that the estimate of this statistic was limited by sample size (*n *=* *86). We caution, however, that phylogenetic signal for pattern presence/absence and UVP were estimated with different metrics, and different sample sizes, and thus are not directly comparable. Pagel's *λ* for UV pattern presence/absence in *Potentilla* was higher than another study that scored flower color discretely (*λ* = 0 in *Erysimum*; Gómez *et al*., 2015) while Blomberg's *K* for quantitative UVP was on a par with another study that evaluated quantitative estimates of floral reflectance (*K* < 0.2 for each of four quantitative floral reflectance estimates in *Iochroma*; Muchhala *et al*., 2014). Although human‐visible flower color is an evolutionary labile trait (Smith & Goldberg, 2015), our results suggest that shared evolutionary history contributes to variation in the presence/absence of floral UV pigmentation patterns.

The presence/absence of UV pattern on flowers was better predicted by specific bioclimatic parameters than geographic ones. In particular, UV‐B irradiance tended to predict the presence/absence of pattern best – species with uniform floral UV absorption tend to grow in areas of higher UV‐B irradiance than those with UV pattern. This finding is consistent with both the protective function of UV absorbance on petals, and the effect of UV‐B irradiance (but not temperature or precipitation) on floral UV pigmentation found for *A. anserina* (Koski & Ashman, 2015a). Our work joins just one other study that tested for an effect of altitude on flower color in a macroevolutionary context. Ogutcen *et al*. (2014) assessed whether reddish pigmentation in *Mimulus* flowers was associated with species restricted to higher elevations but found no such correlation. Their study did not, however, evaluate specific bioclimatic variables that change with altitude. Our results indicate that the marginal positive effect of UV‐B irradiance on UV pattern may result from a tendency for uniformly absorbing species to grow at higher altitudes (Table 3). However, the limited effects of biogeography and bioclimatic variables on the presence/absence of UV pattern may indicate that the trait could be affected by other factors, such as pollinators, or that evolutionary transitions between uniform UV absorption and UV pattern are less common than those among UV‐patterned flowers with varied size of the UV‐absorbing area.

Of the species with UV pattern, there was quantitative variation in the size of the UV bullseye, and for these taxa, there was a strong positive effect of altitude on the extent of UV pigmentation. Higher altitude is associated with both lower temperatures and higher UV irradiance, both of which emerged as significant predictors of UVP variation in the bioclimatic analyses. The positive effect of UV‐B irradiance on UV pigmentation agrees with the marginal effect of UV irradiance found in the analysis with UV pattern scored as a discrete character (this study), and predictions based on intraspecific spatial variation and functional assays of UV pigmentation's function in flowers (Koski & Ashman, 2015a). This work thus extends previous work by supporting the idea that UV irradiance could drive global patterns of UV floral pigmentation diversity among species. The strong negative effect of temperature on UV pigmentation variation among patterned species suggests one of two possibilities. First, UV‐absorbing pigments in flowers (flavonoids in *Potentilla* species; Harborne & Nash, 1984) might ameliorate cold stress. Cold stress can induce the production of flavonoid compounds (Rivero *et al*., 2001), although flavonoids in flowers can ameliorate heat stress as well (Coberly & Rausher, 2003), suggesting that flavonoid production is a generalized response to thermal stress in plants. Alternatively, unobserved pollination dynamics could covary with temperature (and altitude). For example, pollinator community composition (Kearns, 1992) and behaviors in response to floral UV pattern can change with altitude (Koski & Ashman, 2015b). Thus, one cannot rule out these, or other, biotic agents in structuring phenotypic variation. An ideal next step for understanding floral UV pattern diversification will involve evaluating associations between floral phenotypes and both pollination and abiotic factors. Finally, 94% of the taxa included in our PGLS analysis of UV‐patterned species were yellow in the human‐visible spectrum, mirroring the color distribution in the Potentilleae in general. Thus, while we could not rigorously assess the ability of human‐visible flower color to influence the results, comparative analyses (data not shown) show that it did not.

Finally, our PGLS analysis, which included only the 86 UV‐patterned species, consisted of 81 taxa that are yellow in the human‐visible spectrum (94%), and thus potential ecological correlations with human‐visible flower color are unlikely to influence the results obtained for ecological associations with UV pattern. To address whether phenotypic correlations between UV pattern and human‐visible flower color were likely to influence our results, we assessed whether biogeography or abiotic factors were associated with human‐visible flower color (white, yellow, or red) across all 177 species using both phylogenetic ANOVA and PGLS. We found no associations in either analysis (phylANOVAs, all *P *>* *0.44, PGLS, all *P *<* *0.25), but caution that species in Potentilleae are predominantly yellow‐flowered, and thus sample sizes of nonyellow‐flowered species are low. In short, additional analyses support the idea that UV pattern, but not human‐visible flower color, is associated with biogeography and abiotic factors.

Our results bring to the fore the important contribution that abiotic factors can have in structuring diversity in UV floral pigmentation. The fact that UV irradiance has a positive effect on UV absorption in flowers at a macroevolutionary scale confirms and extends similar findings at a microevolutionary level in *A. anserina* (Koski & Ashman, 2015a). Functional tests addressing whether greater UV pigmentation yields higher fitness in cooler temperatures, and whether this is the result of abiotic effect alone or in concert with changes in pollinator assemblages will greatly enhance our understanding of the factors, other than UV irradiance, that may contribute to widespread geographic patterns of floral UV pigmentation variation.

## Author contributions

M.H.K. and T‐L.A. designed the study; M.H.K. collected and analyzed the data; and M.H.K. and T‐L.A. wrote the manuscript.

## Supporting information

Please note: Wiley Blackwell are not responsible for the content or functionality of any supporting information supplied by the authors. Any queries (other than missing material) should be directed to the *New Phytologist* Central Office.

**Fig. S1** Combined ITS, ETS and *trn*L‐F Bayesian consensus tree created in beast with posterior probabilities provided at each node.
**Table S1** Accession information for construction of molecular phylogeny of 183 *Potentilleae* taxa
**Table S2** Floral phenotypes, geographic (altitude, latitude) and bioclimatic (temperature, precipitation and UV‐B irradiance) parameters for 177 species in the Potentilleae tribe
