# Supplementary material for: Macroevolutionary patterns of ultraviolet floral pigmentation explained by geography and associated bioclimatic factors
Source: New Phytol. 2016 Mar 14;211(2):708–18. doi: 10.1111/nph.13921 (PMC6681094; doi:10.1111/nph.13921)
Supplement: Supplementary file 1 — Fig. S1 Combined ITS, ETS and trnL‐F Bayesian consensus tree created in beast with posterior probabilities provided at each node. Table S1 Accession information for construction of molecular phylogeny of 183 Potentilleae taxa Table S2 Floral phenotypes, geographic (altitude, latitude) and bioclimatic (temperature, precipitation and UV‐B irradiance) parameters for 177 species in the Potentilleae tribe [file NPH-211-708-s001.pdf]

New Phytologist

***New Phytologist* Supporting Information Fig. S1 and Tables S1 & S2**

Article title: Macroevolutionary patterns of ultraviolet floral pigmentation explained by geography and associated bioclimatic factors

Authors: Matthew H. Koski and Tia-Lynn Ashman

Article acceptance date: 31 January 2016

The following Supporting Information is available for this article:

Fig. S1 Combined ITS, ETS and *trn*L-F Bayesian consensus tree created in BEAST with posterior probabilities provided at each node.

Table S1 Accession information for construction of molecular phylogeny of 183 *Potentilleae* taxa

Table S2 Floral phenotypes, geographic (altitude, latitude) and bioclimatic (temperature, precipitation and UV-B irradiance) parameters for 177 species in the Potentilleae tribe

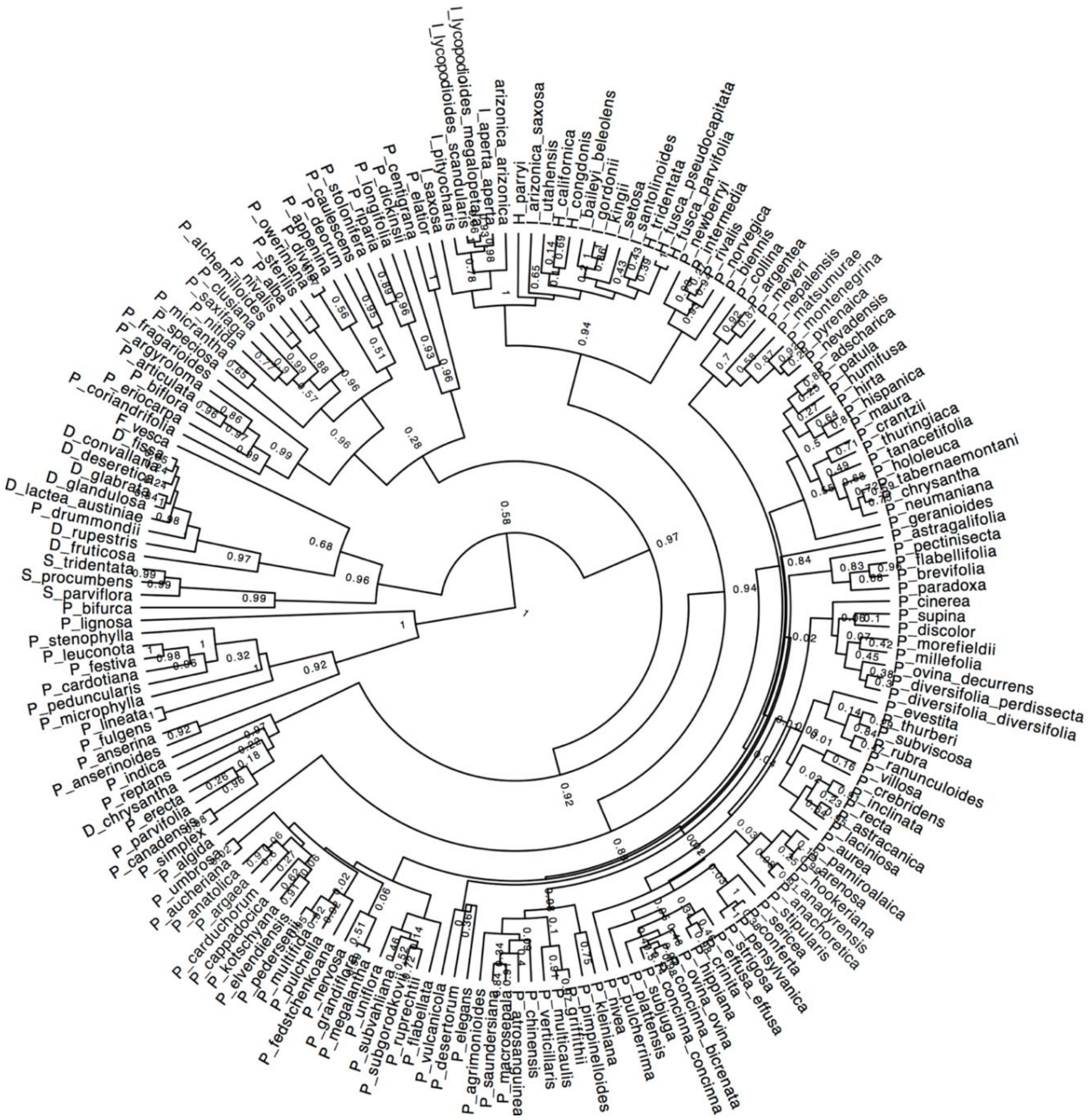

Fig. S1 Combined ITS, ETS and *trn*L-F Bayesian consensus tree created in BEAST with posterior probabilities provided at each node. The following taxa were trimmed from the phylogeny for comparative analyses due to missing phenotypic data: *Ivesia arizonica* var. *saxosa*, *Horkelia fusca* var.

pseudocapitata, *Potentilla humifusa*, *P. neumaniana*, *P. parvifolia*, and *Sibbaldia parviflora*.

Table S1 Accession information for construction of molecular phylogeny of 183 *Potentilleae* taxa. Herbarium voucher, collector, country of origin, year of collection, and Genbank accession number are provided.

Species	Herbarium	Collector	Country	Year	Genbank ITS	Genbank ETS	Genbank *trn*L-F
Argentina anserina							
ITS		T. Eriksson 644	Sweden		U90788.1		
ETS		Eriksson T. TE#153	Sweden			FN421405.1	
trnL-F	HEID 805505		Germany				GQ384662.1
Dasiphora fruticosa							
ITS					GU444027.1		
ETS		T. Eriksson 806				FJ422355.1	
trnL-F		MO 04930363	USA (NM)				GQ384680.1
Drymocallis convallaria							
ITS, ETS, trnL-F	UC1728144	Noel & Patricia Holmgren	USA (ID)	1988	KT985641	KT985769	KT991751
Drymocallis deseretica							
ITS, ETS, trnL-F	UC1583253	Noel & Patricia Holmgren	USA (UT)	1984	KT985642	KT985770	KT991752
Drymocallis fissa							
ITS, ETS, trnL-F	CM496153	F. H. Utech 92-904	USA (CO)	1992	KT985643	KT985771	KT991753
Drymocallis glabrata							
ITS, ETS, trnL-F	UC1583221	N. & P. Holmgren & S. Keller	USA (UT)	1984	KT985644	KT985772	KT991754
Drymocallis lactea var. austiniae							
ITS, ETS	JEPS80322	Vernon H. Oswald & Lowell Ahart	USA (CA)	1996	KT985645	KT985774	
Drymocallis rupestris							
ITS, ETS		M. Lundberg 6			FJ356163	FJ422359.1	
trnL-F	HEID 806644		Spain				GQ384650.1
Duchesnea chrysantha							
ITS, ETS, trnL-F	CM508074	Koji Yonekura	Japan	2002	KT985640	KT985768	KT991750
Duchesnea indica							
ITS		T. Eriksson s.n.	Sweden		U90792.1		
trnL-F		Eriksson s.n.	China				AJ512242.1
Fragaria vesca							

ITS, ETS, trnL-F		Eriksson & Smedmark 43	Sweden		AJ511771.1	FJ422362.1	AJ512232.1
Horkelia californica var. elata							
ITS, ETS, trnL-F	JEPS93802	V. H. Oswald & L. Ahart	USA (CA)	1998	KT985646	KT985775	KT991756
Horkelia congdonis							
ITS, ETS, trnL-F	UC1787123	Michael Honer	USA (CA)	2002	KT985647	KT985776	KT991757
Horkelia fusca var. parviflora							
ITS, ETS, trnL-F	CM322771	M. J. Williams *et al.*	USA (NV)	1983	KT985648	KT985777	KT991758
Horkelia fusca var. pseudocapitata							
ITS, ETS	UC1561186	Ginger V. King	USA (OR)	1987	KT985649	KT985778	
Horkelia parryi							
ITS, ETS	JEPS 102972	M. Foster	USA (CA)	1994	KT985650	KT985779	
Horkelia tridentata							
ITS, ETS	CM485378	F. H. Utech 90-871	USA (CA)	1990	KT985651	KT985780	
trnL-F	UC - Ptl6011	B. Ertter 17986	USA (CA)				GQ384732.1
Ivesia aperta var. aperta							
ITS, ETS	UC1559690	Arnold Tiehm & Jan Nachlinger	USA (NV)	1984	KT985652	KT985781	
trnL-F	UC - Ptl6035	B. Ertter	USA (CA)				GQ384744.1
Ivesia arizonica var. arizonica							
ITS, ETS, trnL-F	RSA508466	Tiehm & Nachlinger 9156	USA (NV)	1984	KT985653	KT985782	KT991759
Ivesia arizonica var. saxosa							
ITS, ETS, trnL-F	UC1559755	A. Tiehm & M. Williams	USA (NV)	1982	KT985654	KT985783	KT991760
Ivesia baileyi var. beneolens							
ITS, ETS, trnL-F	RSA508468	Tiehm & Ertter 9029	USA (NV)	1984	KT985655	KT985784	KT991761
Ivesia gordonii							
ITS, ETS	CM473284	B. Moseley	USA (ID)	1990	KT985656	KT985785	
trnL-F	MO 04635108	Huber 1182	USA (UT)				GQ384725.1
Ivesia kingii							
ITS, ETS, trnL-F		J.L. Reveal *et al.* #4782			FN430787	FN421377	FN561735
Ivesia lycopodioides subsp. megalopetala							
ITS, ETS	RSA131949	Quibell 6509	USA (CA)	1957	KT985657	KT985786	
Ivesia lycopodioides subsp. scandularis							

ITS, ETS, trnL-F	RSA663921	Holmgren & Holmgren 11022	USA (CA)	1985	KT985658	KT985787	KT991762
Ivesia pityocharis							
ITS, ETS, trnL-F	UC1728514	Arnold Tiehm	USA (NV)	1997	KT985659	KT985788	KT991763
Ivesia santolinoides							
ITS, ETS	CM265071	Thorn & DeDecker	USA (CA)	1969	KT985660	KT985789	
trnL-F	UC - Ptl6034	Ertter 16386	USA (CA)				GQ384743.1
Ivesia saxosa							
ITS, ETS	UC1559752	Jim Morefield	USA (CA)	1984	KT985661	KT985790	
trnL-F							GQ384742.1
Ivesia setosa							
ITS, ETS, trnL-F	CM480028	A. Tiehm 12076	USA (NV)	1994	KT985662	KT985791	KT991764
Ivesia utahensis							
ITS, ETS	CM361607	Holmgren & Holmgren	US (UT)	1984	KT985663	KT985792	
trnL-F							GQ384738.1
Potentilla adscharica							
ITS, ETS, trnL-F	E00409739	Davis & Hedge D29539	Turkey	1957	KT985664	KT985793	KT991765
Potentilla agrimonioides							
ITS, ETS, trnL-F	E00663810	Elias, Shelter & Murray 7131	Russia	1984	KT985665	KT985794	KT991766
Potentilla algida							
ITS, ETS, trnL-F	RSA376319	Sojak	Kyrgyzstan	1979	KT985666	KT985795	KT991767
Potentilla alba							
ITS, ETS		Topel M. MA122			FN430774	FN421355.1	
trnL-F	HEID 805513						GQ384664.1
Potentilla alchemilloides							
ITS, ETS, trnL-F		A. Anderberg & A.-L. Anderberg 26			FJ356168	FJ422367	FJ422297
Potentilla anachoretica							
ITS, ETS, trnL-F	E00663806	Korobkov 77-213	Russia	1977	KT985667	KT985796	KT991768
Potentilla anadyrensis							
ITS, ETS, trnL-F	E00663807	Petrovski	Russia	1982	KT985668	KT985797	KT991769
Potentilla anatolica							
ITS, ETS, trnL-F	E00409724	Davis 45494	Turkey	1966	KT985669	KT985798	KT991770
Potentilla anserinoides							

ITS, ETS, trnL-F		Koski	New Zealand	2012	KT985670	KT985799	KT991771
Potentilla apennina var. appenina							
ITS, ETS	E00128311	Akeroyd *et al*. 4200	Italy	1983	KT985671	KT985800	
Potentilla arenosa							
ITS, ETS, trnL-F	Arnold	Deyl & Sojak	Russia	1976	KT985672	KT985801	KT991772
Potentilla argaea							
ITS, ETS, trnL-F	E00409678	Lamond 4760	Azerbaijan	1971	KT985673	KT985802	KT991773
Potentilla argentea							
ITS, ETS, trnL-F		Topel M. MA 143			FN430808.1	FN421387.1	FN561750
Potentilla argyroloma							
ITS, ETS	E00205426	Klein 9223	Iran	1978	KT985674	KT985803	
Potentilla articulata							
ITS, ETS, trnL-F	GB: KGB 324				FN555611.1	FN421410.1	FN666414
Potentilla astracanica							
ITS, ETS, trnL-F	CM250465	Markova	Bulgaria	1971	KT985675	KT985804	KT991774
Potentilla astragalifolia							
ITS, ETS, trnL-F	RSA307559	Elias *et al*. 4781	Russia	1978	KT985676	KT985805	KT991775
Potentilla atrosanguinea							
ITS, ETS, trnL-F		Topel M. MA125			FN430778	FN421372	FN556398
Potentilla aucheriana							
ITS, ETS, trnL-F	E00409752	Soják	Iran	1977	KT985677	KT985806	KT991776
Potentilla aurea							
ITS, ETS	CM359702	Pistarino	Italy	1989	KT985678	KT985807	
trnL-F	HEID 805691	C. Dobeš	Austria				GQ384667.1
Potentilla biennis							
ITS, ETS	RM601177	Dorn 6284	USA (WY)	1995	KT985679	KT985808	
trnL-F	UC - Ptl6074	Ertter 16283	USA (ID)	1998			GQ384775.1
Potentilla biflora							
ITS, trnL-F		Eriksson T. TE#207			FN430826		FN561749
Potentilla bifurca (=*Sibbaldianthae bifurca*)							
ITS, ETS, trnL-F	Gray (A)	Ho *et al*. 1716	China	1996	KT985680	KT985809	KT991777
Potentilla brevifolia							

ITS, ETS	RM579088	Evert 18257	USA (WY)	1989	KT985681	KT985810	
trnL-F	UC - Ptl6041	Ertter 15255	USA (OR)	1996			GQ384748.1
Potentilla canadensis							
ITS, ETS, trnL-F	CM376697	W. A. Zanol 970	USA (PA)	1992	KT985682	KT985811	KT991778
Potentilla cappadocica							
ITS, ETS, trnL-F	E00409794	C. Tobey 982	Turkey	1965	KT985683	KT985812	KT991779
Potentilla cardotiana							
ITS, ETS, trnL-F	Gray (A)	Heng *et al*. 28068	China	2005	KT985684	KT985813	KT991780
Potentilla carduchorum							
ITS, trnL-F	E00081576	Davis & Polunin 24592	Turkey	1954	KT985685		KT991781
Potentilla caulescens							
ITS, ETS, trnL-F		Topel M. MA 133			FN430819.1	FN421379.1	FN556399
Potentilla centigrana							
ITS, ETS, trnL-F	CM349338	Kurosawa & Iketsu	Japan	1988	KT985686	KT985814	KT991782
Potentilla chinensis							
ITS, ETS, trnL-F	CM349851	Zhenhai	China	1988	KT985687	KT985815	KT991783
Potentilla chrysantha							
ITS, ETS, trnL-F		Topel M. MA142			FN430803	FN421385	FN556400
Potentilla cinerea							
ITS, ETS, trnL-F	E00128298	Gardner & Gardner 1982	Spain	1982	KT985688	KT985816	KT991784
Potentilla clusiana							
ITS, ETS, trnL-F		Antonelli A. AA 353			FN430812.1	FN421403.1	FN556401.1
Potentilla collina							
ITS, ETS	Gray (A)	Barta 2001-121	Austria	2001	KT985689	KT985817	
trnL-F	HEID 806679	K. Ujházy	Slovakia	2006			GQ384674.1
Potentilla concinna var. *bicrenata*							
ITS, ETS	RM723139	K.J. Taylor 665	USA (CO)	1997	KT985690	KT985818	
Potentilla concinna var. *concinna*							
ITS, ETS, trnL-F	RM396676	R. Williams	USA (WY)	1987	KT985691	KT985819	KT991785
Potentilla conferta							
ITS, ETS, trnL-F	Arnold	Tsvelev *et al.*	Kazakhstan	1955	KT985692	KT985820	KT991786
Potentilla coriandrifolia							
ITS, ETS, trnL-F	Arnold	Boufford *et al*. 29124	China	1998	KT985693	KT985821	KT991787
Potentilla crantzii							

ITS, trnL-F		Eriksson T. TE 703			FN555609		FN556402
Potentilla crebridens							
ITS, ETS, trnL-F		Eriksen B. BE 569-4			FN430811	FN421356	FN561731
Potentilla crinita							
ITS, ETS, trnL-F	RSA 462447	Gustafson 2461	USA (NM)	1982	KT985694	KT985822	KT991788
Potentilla deorum							
ITS, ETS, trnL-F	E00663821	Archibald 369	Greece	1964	KT985695	KT985823	KT991789
Potentilla desertorum							
ITS, ETS	RSA376320	Sojak	Kyrgyzstan	1979	KT985696	KT985824	
trnL-F	HEID 806610	Berger	Pakistan				GQ384643.1
Potentilla dickinsii							
ITS, ETS, trnL-F		Topel M. MA123			FN430775.1	FN421402.1	FN561727.1
Potentilla discolor							
ITS	PS1079MT01				FJ980389.1		
ETS		Topel M. MA141				FN421396.1	
trnL-F	CM311461	Guilin expedition 70092	China	1984			KT991790
Potentilla diversifolia var. diversifolia							
ITS, ETS, trnL-F	RM729240	Kinter 301	USA (WY)	1994	KT985698	KT985825	KT991791
Potentilla diversifolia var. perdissecta							
ITS, ETS, trnL-F	RM584476	W. Fertig	USA (WY)	1990	KT985697	KT985826	KT991792
Potentilla divina							
ITS		Sojak 17 VII 1984			KJ396294		
Potentilla drummondii							
ITS, ETS		Eriksson T. BGE#1			FN430776.1	FN421357.1	
trnL-F	UC - Ptl6047	Ertter 19403	USA (CA)	2006			GQ384753.1
Potentilla effusa var. effusa							
ITS, ETS	RM563123	B.E. Nelson	USA (WY)	1983	KT985699	KT985827	
trnL-F	UC - Ptl6060	Ertter 12173	USA (CO)	1993			GQ384762.1
Potentilla elatior							
ITS, ETS	E00409317	Sojak	Russia	1983	KT985700	KT985828	
Potentilla elegans							
ITS, ETS		Eriksen B. 1440 1			FN430779	FN421358	
Potentilla elvendensis							
ITS, ETS, trnL-F	E00201630	Rechinger 47328B	Iran	1974	KT985701	KT985829	KT991793

Potentilla erecta							
ITS, trnL-F		Topel M. MA124			FN430780.1		FN556405.1
Potentilla eriocarpa							
ITS, ETS, trnL-F	Arnold	Boufford *et al*. 29172	China	1998	KT985702	KT985830	KT991794
Potentilla evestita							
ITS, ETS, trnL-F	RSA376313	Sojak	Kazakhstan	1979	KT985703	KT985831	KT991795
Potentilla fedstchenkoana							
ITS, ETS, trnL-F	RSA376316	Sojak	Uzbekistan	1979	KT985704	KT985832	KT991796
Potentilla festiva							
ITS, ETS, trnL-F	Arnold	1984 Sino-American Bot. Exp. 869	China	1984	KT985705	KT985833	KT991797
Potentilla flabellata							
ITS, ETS, trnL-F	RSA376317	Sojak	Tajikistan	1981	KT985706	KT985834	KT991798
Potentilla flabellifolia							
ITS, ETS, trnL-F		Topel M. MA 164			FN430810	FN421392	FN556406
Potentilla fragarioides							
ITS, ETS, trnL-F	Gray	Heng *et al*. 28716	China	2005	KT985707	KT985835	KT991799
Potentilla fulgens							
ITS, ETS, trnL-F	Arnold	Boufford *et al*. 28386	China	1998	KT985708	KT985836	KT991800
Potentilla geranioides							
ITS, ETS, trnL-F	E00409804	Davis 10181	Lebanon	1945	KT985709	KT985837	KT991801
Potentilla glandulosa var. reflexa							
ITS, ETS, trnL-F	JEPS96069	Vernon H. Oswald & Lowell Ahart	USA (CA)	1996	KT985710	KT985773	KT991755
Potentilla grandiflora							
ITS, ETS		Topel M. MA 149			FN430806	FN421400	
trnL-F	HEID 805511	C. Dobeš	Italy	2006			GQ384663.1
Potentilla griffithii							
ITS, ETS, trnL-F	Arnold	Zhen-Ju 114043	China	1981	KT985711	KT985838	KT991802
Potentilla hippiana							
ITS, ETS		Eriksson T. BGE#2			FN430801	FN421359	
trnL-F		Eriksson T. TE Bot. Gard. Edinb.					FN556409
Potentilla hirta							

ITS, ETS, trnL-F	RSA460710	Germand & Ledoux 1974	France	1974	KT985712	KT985839	KT991803
Potentilla hispanica							
ITS, ETS, trnL-F	RSA532516	Podlech 47594	Morroco	1989	KT985713	KT985840	KT991804
Potentilla hololeuca							
ITS, ETS, trnL-F	RSA376303	Sojak	Kazakhstan	1979	KT985714	KT985841	KT991805
Potentilla hookeriana							
ITS, ETS	CM457218	W. J. Cody & J. B. McCanse 2156	Canada (NT)	1949	KT985715	KT985842	
trnL-F	UC - Ptl6040	Ertter 12193	USA (CO)	1993			GQ384747.1
Potentilla humifusa							
ITS, ETS, trnL-F	E00409788	Davis 43796A	Turkey	1966	KT985716	KT985843	KT991806
Potentilla inclinata							
ITS, ETS	E00409461	Ekim 783	Turkey	1971	KT985717	KT985844	
trnL-F	HEID 805340	C. Dobeš	France	2006			GQ384658.1
Potentilla intermedia							
ITS, ETS, trnL-F	E00663778	Cantell	Finland	1937	KT985718	KT985845	KT991807
Potentilla kleiniana							
ITS, ETS, trnL-F	CM295009	Seto	Japan	1982	KT985719	KT985846	KT991808
Potentilla kotschyana							
ITS, ETS, trnL-F	E00409661	Davis & Hedge 26865	Turkey	1957	KT985720	KT985847	KT991809
Potentilla laciniosa							
ITS, ETS, trnL-F	E00201631	Hewer 3839	Iran	1976	KT985721	KT985848	KT991810
Potentilla leuconota							
ITS, ETS	CM274520	Bartholomew *et al*. 973	China	1980	KT985722	KT985849	
Potentilla lignosa (=Tylosperma lignosa)							
ITS, ETS	E00409304	Archibald 8045	Turkey	1986	KT985723	KT985850	
trnL-F	W 1990-6892	Sorger & Buchner 82-130-52	Turkey	1982			GQ384793.1
Potentilla lineata							
ITS, ETS trnL-F	Arnold	Boufford *et al*. 30823	China	2004	KT985724	KT985851	KT991811
Potentilla longifolia							
ITS, ETS	RSA544969	Skvortsov	Russia	1989	KT985725	KT985852	
trnL-F	MO	A.K. Skvortsov	Russia	1988			GQ384706.1

	04263053						
Potentilla macrosepala							
ITS, ETS, trnL-F	Arnold	Bartholomew *et al*. 653	China	1984	KT985726	KT985853	KT991812
Potentilla matsumurae							
ITS	CM263168	Onogi	Japan	1979	KT985727		
Potentilla maura							
ITS, ETS	E0063802	Courtney 19	Morocco	1981	KT985728	KT985854	
Potentilla megalantha							
ITS, ETS, trnL-F	CM382796	Deguchi	Japan	1986	KT985729	KT985855	KT991813
Potentilla meyeri							
ITS*, ETS, trnL-F	E00409447	Görk, Hartvig & Strid 24029	Turkey	1984	KT985767	KT985856	KT991814
Potentilla micrantha							
ITS, trnL-F		Eriksson T. TE#149			FN430823		FN561746
Potentilla microphylla							
ITS, ETS, trnL-F		Topel M. MA 144			FN430809	FN421388	FN556412
Potentilla millefolia							
ITS, ETS	JEPS 81657	L.R. Heckard & L. C. & R. Ornduff	USA (CA)	1969	KT985730	KT985857	
trnL-F	UC	B. Ertter 6298	USA (CA)	2006			GQ384765.1
Potentilla montenegrina							
ITS, ETS		Eriksson T. BGE#3			FN430782	FN421361	
trnL-F		Eriksson T. TE Bot. Gard. Edinb.					FN556413
Potentilla morefieldii							
ITS, ETS	JEPS44268	W.L. Jepson	USA (CA)	1917	KT985731	KT985858	
trnL-F	UC	B. Ertter 14489	USA (CA)	1995			GQ384750.1
Potentilla multicaulis							
ITS	CM283809	Ze-Ying	China	1980	KT985732		
trnL-F	MO 05329310	T.N. Ho *et al*. 2325	China	1996			GQ384691.1
Potentilla multifida							
ITS, ETS, trnL-F		Erikssen T. TE705			FN430818	FN421374	FN561734
Potentilla nepalensis							

ITS, ETS, trnL-F		Topel M. MA163			FN430821	FN421390	FN561743
Potentilla nervosa							
ITS, ETS, trnL-F	Arnold	Sojak	Kyrgyzstan	1979	KT985733	KT985859	KT991815
Potentilla neumaniana							
ITS, ETS, trnL-F		Eriksson T. BT#1			FN666607	FN421370	FN556414
Potentilla nevadensis							
ITS, ETS	E00663775	Stocken 238.63	Spain	1963	KT985734	KT985860	
trnL-F	HEID 806627	Ern 140	Spain				GQ384647.1
Potentilla newberryi							
ITS, ETS	UC1587099	Schoolcraft *et al.*	USA (NV)	1991	KT985735	KT985861	
trnL-F	MO 05690792	M.D. Windham & M. Beilstein 2477	USA (NV)	2001			GQ384710.1
Potentilla nitida							
ITS, ETS		Eriksson T. TE825			FN430795	FN421375	
trnL-F	HEID 806879	J. Paule	Slovenia	2006			GQ384679.1
Potentilla nivalis							
ITS, ETS, trnL-F	E00663773	Harrold 553	Spain	1978	KT985736	KT985862	KT991816
Potentilla nivea							
ITS, ETS, trnL-F		Eriksen B. 1672:1			FN430816	FN421371	FN561729
Potentilla norvegica							
ITS, ETS, trnL-F		Eriksen B. BE 1567:1			FN430817	FN421362	FN561730
Potentilla ovina var. decurrens							
ITS, ETS	RM627230	Hartman	USA (WY)	1994	KT985737	KT985863	
trnL-F	UC	B. Ertter 18456	USA (UT)	2004			GQ384767.1
Potentilla ovina var. ovina							
ITS, ETS, trnL-F	RM609206	Evert 6645	USA (WY)	1984	KT985738	KT985864	KT991817
Potentilla oweriniana							
ITS, ETS, trnL-F	E00409404	Davis & Hedge 30397	Turkey	1957	KT985739	KT985865	KT991818
Potentilla pamiroalaica							
ITS, ETS, trnL-F	RSA418933	Sojak	Kazakhstan	1987	KT985740	KT985866	KT991819
Potentilla paradoxa							
ITS, ETS, trnL-F	RM521586	R. L. McGregor	USA (KS)	1980	KT985741	KT985867	KT991820
Potentilla parvifolia							
ITS, ETS	CM366160	Z. Quing-sheng	China	1989	KT985742	KT985868	

Potentilla patula							
ITS, ETS, trnL-F	E00500251	Sukhorukov 112	Russia	2011	KT985743	KT985869	KT991821
Potentilla pectinisecta							
ITS, ETS, trnL-F	RM763737	B.E. Nelson	USA (WY)	2001	KT985744	KT985870	KT991822
Potentilla pedersenii							
ITS, ETS, trnL-F		Eriksen B. 05-24			FN430799	FN421404	FN556415
Potentilla peduncularis var. peduncularis							
ITS, ETS, trnL-F		Topel M. MA173			FN430820	FN421389	FN561742
Potentilla pensylvanica							
ITS, ETS	RM705795	Nelson 31917	USA (WY)	1994	KT985745	KT985871	
trnL-F	UC	B. Ertter 12187	USA (CO)	1993			GQ384774.1
Potentilla pimpinelloides							
ITS, ETS, trnL-F		Topel M. MA139			FN430793	FN421384	FN556417
Potentilla plattensis							
ITS, ETS	RM585069	Fertig 2439	USA (WY)	1990	KT985746	KT985872	
trnL-F	UC	B. Ertter 11429	USA (AZ)	1992			GQ384768.1
Potentilla pyrenaica							
ITS	E00128293	Gardner & Gardner 760	Spain	1980	KT985749		
Potentilla pulchella							
ITS, ETS, trnL-F	E00663796	Halliday H560	Norway	1965	KT985747	KT985873	KT991823
Potentilla pulcherrima							
ITS, ETS	RM585044	Fertig 3447	USA (WY)	1990	KT985748	KT985874	
trnL-F	MO05033056	J.S. Miller 8268	USA (CO)	1994			GQ384695.1
Potentilla ranunculoides							
ITS, trnL-F	CM224183	Antipovitch	Mexico	1928	KT985750		KT991824
Potentilla recta							
ITS, ETS, trnL-F		Eriksson T. BT#2			FN430784	FN421393	FN556419
Potentilla reptans							
ITS, ETS, trnL-F		Topel M. MA131			FN430815	FN421368	FN561728
Potentilla riparia							
ITS, ETS	CM294202	Murata *et al.*	Japan	1976	KT985751	KT985875	
Potentilla rivalis							
ITS*, ETS	CM457492	Krivda	Canada	1956	KT985766	KT985876	

			(MB)				
trnL-F		Topel 310					FR872992
Potentilla rubra							
ITS, ETS, trnL-F	CM224184	Antipovitch	Mexico	1927	KT985752	KT985877	KT991825
Potentilla ruprechtii							
ITS, ETS, trnL-F	E00409744	Sojak	Russia	1983	KT985753	KT985878	KT991826
Potentilla saundersiana							
ITS, ETS, trnL-F	CM283802	Zhen-Ju	China	1981	KT985754	KT985879	KT991827
Potentilla saxifraga							
ITS, ETS, trnL-F	E00663795	Charpin 13914	France	1977	KT985755	KT985880	KT991828
Potentilla sericea							
ITS, ETS, trnL-F	Arnold	Nabrob 857	Mongolia	1926	KT985756	KT985881	KT991829
Potentilla simplex							
ITS, ETS	CM457495	S. Brisson & C. Hamel 12,277	Canada (QC)	1967	KT985757	KT985882	
trnL-F	MO 05171738	D. Rowan 94-15	USA (MO)	1994			GQ384717.1
Potentilla speciosa							
ITS, ETS, trnL-F	RSA352162	Archibald 6847	Turkey	1985	KT985758	KT985883	KT991830
Potentilla stenophylla							
ITS		Eriksson & Vretblad TE763			AJ511780		
ETS, trnL-F		Eriksson T. GBT#1				FN421381	FN561738
Potentilla sterilis							
ITS, ETS, trnL-F		Eriksson T. TE734			FN555612	FN421376	FN561732
Potentilla stipularis							
ITS	E00663784	Argent	Greenland	1962	KT985759		
Potentilla strigosa							
ITS, ETS, trnL-F	Arnold		Russia	1949	KT985760	KT985884	KT991831
Potentillla stolonifera							
ITS, ETS, trnL-F		Eriksen B. 1382:1			FN430814	FN421363	FN556420
Potentilla subgorodkovii							
ITS, ETS, trnL-F	RM521873	Lackschewitz 10549	USA (MO)	1983	KT985761	KT985885	KT991832
Potentilla subjuga							

ITS, ETS	CM298016	Siplivinsky	USA (CO)	1982	KT985762	KT985886	
trnL-F	UC	B. Ertter 12150	USA (CO)	1993			GQ384776.1
Potentilla subvahliana							
ITS, ETS, trnL-F		Eriksen B. 931-3-05			FN430783	FN421364	FN556421
Potentilla subviscosa							
trnL-F	UC	Ertter 11870	USA	1993			GQ384779
Potentilla supina							
ITS, ETS	E00409603	Hewitt 272	Turkey	1970	KT985763	KT985887	
trnL-F	HEID 806484	Mayr A.	Austria	2005			GQ384641
Potentilla tabernaemontani							
ITS, ETS		Eriksson T. SG#1			FN555608	FN421365	
trnL-F		Eriksson T. Spont. GBG					FN556466
Potentilla tanacetifolia							
ITS, ETS, trnL-F		Eriksson T. ex. Leipzig-98			FN430797	FN421366	FN556422
Potentilla thurberi							
ITS, ETS, trnL-F		Topel M. MA138			FN430792	FN421383	FN561740
Potentilla thurangiaca							
ITS, ETS, trnL-F		Topel M. MA119			FN430777	FN421406	FN556423
Potentilla umbrosa							
trnL-F		HEID 806401	Ukraine				GQ384633.1
Potentilla uniflora							
ITS, ETS, trnL-F		Eriksen B. 271-4-05			FN430785	FN421367	FN556425
Potentilla verticillaris							
ITS, ETS, trnL-F	RSA376301	Sojak	Mongolia	1965	KT985764	KT985888	KT991833
Potentilla villosa							
ITS, ETS, trnL-F		Topel M. MA127			FN430786	FN421369	FN556426
Potentilla vulcanicola							
trnL-F	MO 04627822	V.V. Yakubov	Russia	1990			GQ384699
Sibbaldia procumbens							
ITS, ETS							
Sibbaldia parviflora							
ITS, ETS		M. Lundberg 4			FJ356174	FJ422374	
Sibbaldiopsis tridenta							

ITS, ETS, trnL-F	CM524448	B. L. Isaac & C. F. Chuey 21231	Canada (NL)	2011	KT985765	KT985889	KT991834

* ITS1-2 only

Table S2 Floral phenotypes, geographic (altitude, latitude) and bioclimatic (temperature, precipitation and UV-B irradiance) parameters for 177 species in the Potentilleae tribe used for phylogenetic comparative analyses

Species	UV Proportion	UV Pattern (presence/absence)	Human-Visible Color	Average Altitude (m)	Average Latitude	Mean Annual Temperature (°C)	Mean Annual Precipitation (mm)	Mean Annual UV-B Irradiance (J m^{-2} d^{-1})
D_chrysantha	0.00	0	Y	368.8345	33.9924	14.2439	1775.6901	2843.3573
D_convallaria	1.00	1	W	1283.6944	44.5906	6.2628	504.0556	2490.1128
D_deseretica	1.00	1	W	2951.2000	40.8006	0.9400	742.2000	3449.4502
D_fissa	1.00	1	Y	2092.4643	41.1661	5.6758	457.7738	2982.7583
D_fruticosa	1.00	1	Y	811.5979	51.9468	4.7864	682.1280	2149.0180
D_glabrata	1.00	1	Y	1555.1200	44.6907	4.4180	591.0400	2471.3451
D_glandulosa	1.00	1	W	1276.3294	42.8403	7.1101	839.0493	2673.8587
D_lactea_austiniae	1.00	1	Y	1666.3696	41.0135	6.6008	1012.0000	2932.4145
D_rupestris	1.00	1	W	864.4116	46.0117	7.1101	839.0493	2673.8587
F_vesca	1.00	1	W	194.1500	56.0184	6.7024	780.6938	1532.9979
H_californica	1.00	1	W	457.0341	38.0670	13.0468	854.8780	3077.2460
H_congdonis	1.00	1	W	2382.1538	37.5922	5.4397	441.2308	3700.7351
H_fusca_parvifolia	0.00	0	W	1963.8547	42.6571	5.1513	536.3143	3228.7260
H_parryi	1.00	1	W	647.2143	38.2811	13.9485	819.8571	3251.3862
H_tridentata	1.00	1	W	1333.9412	39.8334	9.2735	1120.3382	3057.0796
I_aperta_aperta	1.00	1	Y	1800.8182	39.5963	7.2621	492.0909	3174.5272
I_arizonica_arizonica	0.58	0	Y	1944.6667	36.4468	10.0431	249.3333	3480.3000
I_baileyi_beleolens	0.19	0	W	1805.4000	42.1938	5.7437	353.1200	2807.8196
I_gordonii	0.43	0	Y	2489.1940	41.3924	2.2790	727.2090	3115.4676
I_kingii	1.00	1	W	1834.0169	39.0879	8.2461	263.4576	3255.5959
I_lycopodioides_megalopetala	0.47	0	Y	3019.3538	37.3310	2.1056	692.4615	3929.9249
I_lycopodioides_scandularis	0.44	0	Y	3380.5510	37.4578	0.3181	602.7347	3750.2706
I_pityocharis	1.00	1	W	2387.2000	38.8737	4.9358	522.0000	3330.8667
I_santolinoides	1.00	1	W	2149.2830	37.2932	6.8465	757.3019	3625.1843
I_saxosa	0.16	0	Y	1810.9048	36.1247	10.3851	350.4286	3678.7331
I_setosa	0.41	0	Y	2132.8909	39.7866	6.1401	315.2000	3097.7075

I_utahensis	1.00	1	W	2994.8095	40.4195	1.0296	807.5714	3209.0344
P_adscharica	0.45	0	Y	1557.0000	41.5085	6.4683	617.8000	2654.5054
P_agrimonioides	1.00	1	Y	3985.5532	36.3232	-1.0807	225.2979	4878.7831
P_alba	1.00	1	W	300.4391	50.5471	8.3472	686.6563	1714.3814
P_alchemilloides	1.00	1	W	1508.4286	42.6442	6.3993	1123.1143	2457.8334
P_algida	1.00	1	Y	1900.0000	42.6617	3.8333	423.0000	2807.0061
P_anachoretica	1.00	1	Y	495.5000	68.3746	-14.4833	217.1250	1152.7622
P_anadyrensis	1.00	1	Y	248.0000	65.5628	-10.5097	304.5000	1211.7348
P_anatolica	0.75	0	Y	2877.0000	37.3091	3.7500	620.0000	3399.4678
P_anserina	0.48	0	Y	74.2222	54.0593	8.4084	781.9585	1485.9608
P_anserinoides	0.34	0	Y	267.2766	42.9634	10.8422	1401.5532	2434.5828
P_appenina	1.00	1	W	1482.0000	42.3504	6.9875	935.5000	2439.9268
P_arenosa	0.99	1	Y	412.7561	67.0862	-7.3888	291.2073	1169.5829
P_argaea	0.58	0	Y	3100.6667	40.4600	-1.4167	664.0000	3064.0303
P_argentea	0.51	0	Y	142.4791	56.7657	6.6055	695.6518	1382.6118
P_argyroloma	1.00	1	Y	1665.1409	34.8372	14.9861	122.3333	3882.2860
P_articulata	0.21	0	Y	4005.2222	29.0681	2.7074	886.4444	5582.6241
P_astracanica	0.12	0	Y	874.4286	43.1924	9.1792	727.5714	2368.1500
P_astragalifolia	0.97	1	Y	1770.0000	49.6708	-4.7417	241.5000	2369.7201
P_atrosanguinea	1.00	1	R	1477.3929	32.6558	6.5014	838.3750	5166.4532
P_aucheriana	0.45	0	Y	3174.3333	36.7688	4.1889	420.3333	3604.5683
P_aurea	0.73	0	Y	1255.1749	46.4709	5.3026	1106.8296	2039.2919
P_biennis	0.01	0	Y	1574.3876	41.9727	7.0350	389.6746	2887.4822
P_biflora	0.28	0	Y	1384.6887	59.4701	-6.8098	324.6462	2012.1579
P_bifurca	0.57	0	Y	3809.3684	35.0537	-0.3498	446.7895	5035.1559
P_brevifolia	0.64	0	Y	2215.4783	44.1481	1.3875	621.2174	2638.7124
P_canadensis	1.00	1	Y	131.6310	40.9159	10.1477	1161.7380	2383.7806
P_cappadocica	0.35	0	Y	1481.5000	40.8200	7.5521	524.5000	2671.3950
P_cardotiana	0.96	1	Y	3257.8400	27.5557	8.0625	989.0400	5998.3897
P_carduchorum	0.63	0	Y	2434.5000	38.1836	6.0146	745.0000	3186.3348
P_caulescens	1.00	1	W	993.8045	41.5310	10.5225	747.0652	2641.3479
P_centigrana	0.30	0	Y	949.6988	35.0516	10.2377	1595.3855	2836.9734
P_chinensis	0.14	0	Y	1032.6429	34.1665	12.4156	1273.3095	3473.2727
P_chrysantha	0.93	0	Y	3275.7000	40.0608	-0.3254	362.5000	4172.6321

P_cinerea	0.79	0	Y	1067.1343	41.4011	10.2425	637.5821	2672.1209
P_clusiana	1.00	1	Y	1287.1724	47.7571	3.7172	1309.9655	1875.8471
P_collina	1.00	1	Y	283.2319	50.1739	8.7789	700.6014	1754.3306
P_concinna_bicrenata	1.00	1	Y	2558.3243	38.2259	4.0970	432.5135	3667.4543
P_concinna_concinna	1.00	1	Y	1332.6486	47.4296	4.0528	423.0270	2187.3481
P_conferta	1.00	1	Y	2163.5556	36.5491	3.5958	555.5000	4091.1134
P_coriandrifolia	1.00	1	W	3527.1515	27.6605	6.5352	895.6364	5977.4417
P_crantzii	0.78	0	Y	553.0437	62.2377	2.0575	910.0378	1199.7998
P_crebridens	0.95	0	Y	837.3182	67.9873	-11.7016	240.2500	1184.3120
P_crinita	1.00	1	Y	2357.0256	35.9927	6.6688	433.1282	3526.6039
P_deorum	1.00	1	W	2177.5000	40.0915	3.6188	865.5000	2581.4678
P_desertorum	0.65	0	Y	3706.0725	35.6919	1.0754	416.8696	4937.0143
P_dickinsii	0.17	0	Y	951.4267	36.1222	9.0769	1749.9067	2625.4658
P_discolor	0.52	0	Y	193.7500	28.8543	18.1439	1740.4063	3278.5205
P_diversifolia_diversifolia	1.00	1	Y	2203.3941	47.2641	0.0264	679.6626	2514.7943
P_diversifolia_perdissecta	1.00	1	Y	2646.8690	44.2527	-0.4770	574.4643	2973.4760
P_divina	1.00	1	R	2760.0000	43.1901	-0.1417	918.0000	3070.9558
P_drummondii	1.00	1	Y	1872.6335	43.6251	3.7976	1084.8054	2705.8115
P_effusa_effusa	0.68	0	Y	2264.0500	46.8185	2.8646	299.5000	2320.3143
P_elatior	1.00	1	W	1021.0000	41.8000	9.0583	713.0000	2634.8564
P_elegans	0.56	0	Y	777.0192	66.2982	-9.4968	303.3654	1119.1025
P_elvendiensis	0.58	0	Y	1656.6667	32.1354	15.1250	237.0000	4507.7841
P_erecta	0.15	0	Y	172.5543	56.2088	6.8622	847.2996	1382.3212
P_eriocarpa	0.23	0	Y	3800.8704	29.3774	4.2979	740.3889	5779.8017
P_evestita	1.00	1	Y	2039.5263	51.3707	-2.8042	283.8421	2590.3799
P_fedstchenkoana	0.91	0	Y	2216.0000	41.2198	4.0708	755.0000	3168.9124
P_festiva	0.95	1	Y	3473.2308	28.5868	6.5676	891.6154	5465.4873
P_flabellata	0.59	0	Y	3646.6667	35.7781	0.2069	807.3333	4787.2170
P_flabellifolia	1.00	1	Y	1789.8600	45.0015	3.0847	1320.5100	2420.0647
P_fragarioides	0.51	0	Y	387.7186	35.3155	12.9395	1640.4731	2716.9154
P_fulgens	1.00	1	Y	2943.8571	27.2261	9.3241	979.0714	5129.9498
P_geranioides	0.50	0	Y	1873.0000	33.3503	10.1500	910.0000	3850.7129
P_grandiflora	0.66	0	Y	559.0030	62.9038	1.8382	907.2356	1182.9156
P_griffithii	1.00	1	Y	2994.5526	27.7452	9.2018	893.0000	5538.1617

P_hippiana	0.92	0	Y	2444.3125	38.7009	4.5338	528.8542	3432.7335
P_hirta	0.49	0	Y	809.8090	41.2523	11.9892	724.3596	2679.6758
P_hispanica	0.49	0	Y	1759.2381	34.5543	10.9411	585.2857	3544.0781
P_hololeuca	0.99	1	Y	2407.6000	36.4605	8.4100	117.0000	4214.7177
P_hookeriana	0.84	0	Y	683.1096	63.0000	-6.7710	324.2603	1427.5491
P_inclinata	0.16	0	Y	362.9252	49.0268	8.9079	756.9150	1849.3645
P_indica	0.26	0	Y	259.8696	39.7027	13.1540	1080.2446	2679.5929
P_intermedia	0.28	0	Y	98.0166	57.3330	6.2494	707.4131	1349.6263
P_kleiniana	0.44	0	Y	1230.0000	29.1654	15.2538	1426.5636	4457.8751
P_kotschyana	0.11	0	Y	3849.5000	49.6708	0.4292	160.5000	4657.1421
P_laciniosa	0.21	0	Y	405.7143	41.5519	12.4256	564.5714	2560.1026
P_leuconota	0.87	0	Y	2818.5738	24.9487	8.6188	2780.2131	4220.0138
P_lignosa	1.00	1	W	2324.4444	37.0264	8.3468	402.1111	3861.5644
P_lineata	1.00	1	Y	2267.8333	27.6301	12.5991	1076.3333	4880.9076
P_longifolia	1.00	1	Y	1831.3750	47.5767	0.4203	511.1250	2339.2684
P_macrosepala	0.83	0	Y	3246.5000	27.5636	8.0045	914.9167	5983.9360
P_matsumurae	1.00	1	Y	1980.8718	30.5683	7.4011	2569.3077	3218.3619
P_maura	0.50	0	Y	2138.5714	33.6559	9.4610	682.0000	3756.0105
P_megalantha	1.00	1	Y	185.6667	47.4997	6.8491	1086.6667	1817.0192
P_meyeri	0.43	0	Y	2618.3333	37.5680	3.1653	613.3333	2980.2444
P_micrantha	1.00	1	W	960.1022	44.2091	8.6879	885.5376	2302.8247
P_microphylla	1.00	1	Y	4012.4091	29.2008	3.6900	671.6364	6013.3849
P_millefolia	1.00	1	Y	1641.4400	41.4420	6.5688	597.1200	2911.9377
P_montenegrina	0.10	0	Y	213.0000	50.7556	9.5333	893.0000	1597.1980
P_morefieldii	1.00	1	Y	3101.4444	37.5728	1.9019	531.6667	3591.8478
P_multicaulis	0.78	0	Y	3339.3636	36.5300	0.8383	512.7273	4642.7604
P_multifida	1.00	1	Y	3312.5379	41.9946	-1.2649	289.2348	4301.2992
P_nepalensis	0.13	0	R	2362.8824	33.4982	10.7392	1027.0000	4540.3648
P_nervosa	1.00	1	Y	4170.3333	35.7967	-1.7972	289.0000	4993.2747
P_nevadensis	0.56	0	Y	1584.6429	38.1148	10.0490	723.0714	3052.0854
P_newberryi	1.00	1	W	1493.8125	42.3170	7.2321	322.0625	2836.8046
P_nitida	1.00	1	R	1621.6250	46.5105	3.6971	1067.1250	2098.6430
P_nivalis	1.00	1	W	1695.7184	42.7652	5.4858	1108.3103	2431.9471
P_nivea	1.00	1	Y	904.7716	63.9689	-1.8967	773.6771	1255.3436

P_norvegica	0.15	0	Y	162.0062	58.9839	4.7513	676.4569	1303.3196
P_ovina_decurrens	1.00	1	Y	2925.1014	41.1609	0.9205	565.4928	3264.7728
P_ovina_ovina	1.00	1	Y	2545.1429	42.0750	1.8985	481.1786	3003.0013
P_oweriniana	1.00	1	R	2216.0000	41.2198	4.0708	755.0000	2771.8359
P_pamiroalaica	1.00	1	Y	4033.6250	36.8243	-1.6188	195.3750	4806.1430
P_paradoxa	0.30	0	Y	1563.3542	42.8646	7.1720	303.1875	2765.4555
P_patula	0.23	0	Y	491.0000	48.2820	7.3385	624.7500	1957.9250
P_pectinisecta	1.00	1	Y	2032.3247	38.8284	6.5835	481.3074	3334.1907
P_pedersenii	1.00	1	Y	32.0000	73.8317	-12.6958	162.0000	1243.2012
P_peduncularis	0.65	0	Y	3433.3158	28.8235	6.6171	894.4211	5882.9183
P_pensylvanica	1.00	1	Y	1367.5991	49.4748	1.1416	488.1636	2291.0743
P_pimpinelloides	0.15	0	Y	1567.6667	42.8961	6.2764	414.0000	3046.5906
P_plattensis	1.00	1	Y	2422.8571	40.7925	2.7863	514.4286	3460.5239
P_pulchella	1.00	1	Y	143.2281	76.4933	-8.0676	246.4063	1199.6075
P_pulcherrima	1.00	1	Y	2613.5411	39.4887	3.0391	548.2415	3493.4076
P_pyrenaica	0.73	0	Y	1754.5566	42.2875	5.7359	1077.9434	2483.6508
P_ranunculoides	0.99	1	Y	2499.3889	21.0702	13.3833	1054.5000	5259.2163
P_recta	0.04	0	Y	239.5287	48.4755	8.7644	760.2334	1769.1565
P_reptans	0.27	0	Y	102.5299	51.8717	9.2889	740.8509	1615.7547
P_riparia	1.00	1	Y	335.5455	34.8057	13.4735	1757.3636	2778.1774
P_rivalis	0.06	0	Y	1067.0688	41.6367	8.9399	523.1688	2830.6675
P_rubra	0.81	0	R	2626.5769	21.9691	12.7829	1041.0000	5330.5728
P_ruprechtii	0.66	0	Y	2035.2000	41.1091	4.6417	675.2000	2772.6994
P_saundersiana	0.84	0	Y	4395.2770	30.8105	0.6070	460.6351	5842.6272
P_saxifaga	1.00	1	W	307.2000	47.9674	9.8792	740.2000	1910.3325
P_sericea	1.00	1	Y	2733.2222	43.1963	-1.6324	387.6667	3659.1543
P_simplex	1.00	1	Y	136.6053	41.9884	9.1633	1145.9887	2294.7505
P_speciosa	1.00	1	W	1346.1538	38.5626	9.5155	912.0385	2835.9079
P_stenophylla	0.45	0	Y	4067.3721	29.4850	2.7206	738.5116	5826.5260
P_sterilis	1.00	1	W	198.7015	50.5923	9.4645	865.9096	1647.4137
P_stipularis	1.00	1	Y	374.6250	66.7125	-9.4547	272.6250	1185.7192
P_stolonifera	0.44	0	Y	1135.5000	39.3297	6.5729	1904.9000	2413.5596
P_strigosa	1.00	1	Y	997.7500	44.9660	3.4563	279.5000	2382.2899
P_subgorodkovii	1.00	1	Y	1452.7059	61.6697	-6.4404	474.9412	1339.3508

P_subjuga	1.00	1	Y	3393.1429	38.9453	-0.9542	725.2857	3987.5517
P_subvahliana	0.92	0	Y	541.2000	66.3256	-8.2292	314.0818	1113.4425
P_subviscosa	0.69	0	Y	2284.7143	33.3479	9.4976	602.8571	3810.4922
P_supina	0.25	0	Y	266.8631	48.0818	9.7528	693.8013	1915.8135
P_tabernaemontani	0.31	0	Y	291.1273	51.6482	8.1867	764.1650	1647.5138
P_tanacetifolia	0.86	0	Y	1253.7500	47.5424	2.0917	446.2500	2426.1757
P_thurberi	0.32	0	R	2048.5413	31.1923	11.7808	670.6881	4070.7961
P_thuringiaca	0.49	0	Y	170.8110	60.1905	4.5570	687.7426	1222.4585
P_umbrosa	0.55	0	Y	1790.0000	41.1227	5.4938	676.5000	2583.5867
P_uniflora	0.93	0	Y	1340.1961	58.6700	-5.1583	554.1078	1598.7334
P_verticillaris	1.00	1	Y	95.0000	50.6304	9.7458	842.0000	1611.6482
P_villosa	0.69	0	Y	499.4671	55.9270	2.3894	1313.3114	1276.5513
P_vulcanicola	1.00	1	Y	503.6585	63.9911	-5.4451	429.1220	1072.5685
S_procumbens	1.00	1	Y	878.1822	62.0480	0.3442	1106.7764	1229.6178
S_tridentata	1.00	1	W	287.4793	47.8568	3.6254	1036.5576	1873.7226
